# Referential Chains Reveal Predictive Processes and Form-to-Function Mapping: An Electroencephalographic Study Using Naturalistic Story Stimuli

**DOI:** 10.3389/fpsyg.2021.623648

**Published:** 2021-08-19

**Authors:** Ingmar Brilmayer, Petra B. Schumacher

**Affiliations:** Department of German Language and Literature I–Linguistics, University of Cologne, Cologne, Germany

**Keywords:** prominence, reference, prediction, form-to-function mapping, naturalistic stimuli, P300, N400

## Abstract

In discourse pragmatics, different referential forms are claimed to be indicative of the cognitive status of a referent in the current discourse. Referential expressions thereby possess a double function: They point back to an (existing) referent (form-to-function mapping), and they are used to derive predictions about a referent’s subsequent recurrence in discourse. Existing event-related potential (ERP) research has mainly focused on the form-to-function mapping of referential expression. In the present ERP study, we explore the relationship of form-to-function mapping and prediction derived from the antecedent of referential expressions in naturalistic auditory language comprehension. Specifically, the study investigates the relationship between the form of a referential expression (pronoun vs. noun) and the form of its antecedent (pronoun vs. noun); i.e., it examines the influence of the interplay of predictions derived from an antecedent (forward-looking function) and the form-to-function mapping of an anaphor (backward-looking function) on the ERPs time-locked to anaphoric expressions. The results in the time range of the P300 and N400 allow for a dissociation of these two functions during online language comprehension.

## Introduction

It is a common observation in pragmatic research on discourse structure that different referential forms are indicative of the cognitive status of a referent in the mind of the speaker, as well as of the cognitive status that a speaker assumes in a hearer (e.g., Prince, 1981; Givón, 1983; Ariel, 1990; Gundel et al., 1993). Accordingly, specific referential forms, such as personal pronouns, demonstratives, full noun phrases, or names, can be seen as pointers to the cognitive status of a discourse referent. In the literature, various approaches to discourse structure include a notion of this cognitive status as a key component, such as *salience, attentional focus, accessibility, referential activation*, *givenness*, or *prominence* (Chafe, 1976; Grosz and Sidner, 1986; Ariel, 1990; Gundel et al., 1993; Grosz et al., 1995; Lambrecht, 1996; Chiarcos et al., 2011; Falk, 2014; von Heusinger and Schumacher, 2019). Here, we follow the prominence account to the cognitive status of referents (see Himmelmann and Primus, 2015; von Heusinger and Schumacher, 2019, for details) which aims at a precise characterization of the cognitive status of discourse referents on the basis of linguistic prominence features (e.g., thematic role, syntactic function, and definiteness). In its discourse-pragmatic formulation (von Heusinger and Schumacher, 2019), the prominence account rests on three basic definitions: (1) Prominence is a relational property that singles out one element from a set of elements of equal rank (e.g., two discourse referents) (2) it shifts in time, e.g., the prominence status of a referent can change, while a discourse unfolds, and (3) prominent referents are structural attractors, i.e., they attract linguistic operations, such as serving as perspectival anchors or licensing more referential variation. In the present event-related potential (ERP) study, we focus on criteria (1) and (3). Specifically, we investigate the relationship between the form of a referential expression (pronoun vs. noun) and the form of its antecedent (pronoun vs. noun), i.e., we examine the contribution of referential chains, i.e., the interplay of antecedent and anaphor during referential processing.

A widely employed indicator for the prominence of referents is the referential form that is used to refer to them. For example, personal pronouns (or other phonetically light forms) with anaphoric function have been claimed to refer to the most prominent entity in the current discourse, while phonetically richer forms, such as full noun phrases, are used to refer to less prominent or newly introduced referents (e.g., Givón, 1983; Ariel, 1990; Gundel et al., 1993; von Heusinger and Schumacher, 2019). In this sense, personal pronouns select the most prominent discourse referent, which is singled-out from all other (less prominent) discourse referents (definition 1). A well-known consequence of this form-to-function mapping of referential expressions is the so-called repeated name penalty (Gordon et al., 1993, 1999; Gordon and Hendrick, 1997; Gelormini-Lezama and Almor, 2011; Almor et al., 2017). Almor et al. (2017), for instance, tested a prominent referent that was introduced by a proper name and was rementioned with the same name again instead of a personal pronoun (e.g., “John went to the store. John/He wished to buy some candy.”; Almor et al., 2017, p. 56) and found that this repetition results in processing costs. Importantly, the authors found that a repeated name with a non-prominent referent, for instance a conjoined noun phrase (e.g., “John and Mary went to the store. John/He wished to buy some candy.”; Almor et al., 2017, p. 56), did not elicit a repeated name penalty, exemplifying the critical role of prominence information in the establishment of coreference (see also for other non-prominent antecedents, like objects, Gordon et al., 1993; Almor, 1999; Burkhardt and Roehm, 2007; Almor and Eimas, 2008).

Moreover, prominent referents allow for more variability in the referential expressions that can be used to refer to them, i.e., they are *structural attractors* (definition 3). Gundel et al. (1993) already note that a prominent referent (a referent “in focus” in Gundel et al.’s terminology) is preferably referred to by an unstressed personal pronoun or a zero marked expression, yet it might also be referred to by a definite description or a proper name. Yet, less accessible referents can only be referred to by a more limited set of referential expressions. For instance, a newly introduced referent can (usually) not be introduced by a definite description, but must be introduced by an indefinite description. Here, we subsume this line of research under the term *backward-looking function of referential expressions* (cf. Givón, 1983): It focuses on the mapping of the referential form of an anaphor to referents in a discourse model (form-to-function mapping). However, as von Heusinger and Schumacher (2019) argue in accordance with Givón (1983), referential expressions also possess a *forward-looking* or *discourse structuring potential*: Prominent referents have a higher probability to recur in subsequent discourse, preferably with a personal pronoun or other phonetically light expressions (Givón, 1983; for behavioral and electrophysiological evidence, see Brocher and von Heusinger, 2018; Fuchs and Schumacher, 2020). In other words, prominent referents have a stronger influence on the way a discourse unfolds, than non-prominent referents; i.e., they attract linguistic operations (definition 3) at the discourse level.

In the present research, we explore the relationship of the form of a referential expression and the form of its antecedent during online language comprehension using ERP. For this purpose, we analyzed electroencephalographic (EEG) data originally recorded by Brilmayer et al. (2019), who used a German audio book recording of The Little Prince by de Saint-Exupéry (2012) as experimental stimulus. The recording is annotated for a wide range of linguistic features (e.g., syntactic function, thematic role, case, number, part-of-speech, and referential features) and physical properties (e.g., pitch contour and speech envelope) but also for the text-analytic measures proposed by Givón (1983). Here, we contrast referential chains with different referential forms. Based on their particularly strong prominence contrast, we chose to contrast anaphoric nouns and pronouns with noun or pronoun antecedents, resulting in four conditions: pronouns with a pronoun antecedent [pronoun-pronoun chain (1)], pronouns with a noun antecedent [noun-pronoun chain (2)], nouns with a noun antecedent [noun-noun chain (3)], and nouns with a pronoun antecedent [pronoun-noun chain (4)].

1. **She** (the flower) adjusted her petals one by one. **She** did not wish to go out into the world all rumpled, like the field poppies (The Little Prince, chapter 8).
2. But **the flower** was not satisfied to complete the preparations for her beauty in the shelter of her green chamber. **She** chose her colors with the greatest care (The Little Prince, chapter 8).
3. I have serious reason to believe that the planet from which the little prince came is **the asteroid known as B−612. This asteroid** has only once been seen through the telescope (The Little Prince, chapter 4).
4. But he was in Turkish costume, and so nobody would believe what he said. Grown−ups are like that. Fortunately, however, for the reputation of Asteroid B−612, a Turkish dictator made a law that his subjects, under pain of death, should change to European costume. So in 1920 **the astronomer** gave his demonstration all over again, dressed with impressive style and elegance (The Little Prince, chapter 4).

Based on the literature on referential form and prominence (e.g., Givón, 1983; Gundel et al., 1993; Arnold, 1998; Kehler et al., 2008; von Heusinger and Schumacher, 2019), we assume that referents of pronouns with pronoun antecedent are the most prominent referents in our current comparison, because both the antecedent and anaphoric expression clearly mark their referent as prominent. They are followed by pronoun anaphors with noun antecedent, since nouns mark a referent as less prominent than pronouns but having a pronoun anaphor enhances the prominence status of the referent (e.g., Givón, 1983). Regarding noun anaphors, pronoun-noun chains are the most unlikely type of the four present referential chains with regard to the prominence information provided by the anaphor and antecedent: In this case, a referent established as prominent (reference *via* pronoun) is continued with an expression marking it non-prominent, which constitutes a discourse structural mismatch (as long as the referent is still accessible in memory). Note however that across a longer narrative, a pronoun-noun chain is likely in cases where the referent must be reactivated after a longer sequence without any mention. Noun anaphors with noun antecedent, by contrast, are very common, for instance in referential chains consisting of an indefinite noun phrase antecedent and a definite noun anaphor or to avoid referential ambiguity. In summary, we propose the following prominence ranking for the four referential chains under examination: pronoun-pronoun > noun-pronoun > noun-noun > pronoun-noun. Before we move on to the discussion of previous ERP studies and our experimental hypothesis, we would like to elaborate on the neurobiological understanding underlying our assumptions and the interpretation of our results.

In the present manuscript, we base our hypotheses and the discussion of the results on a predictive coding account to ERPs (Friston, 2005), which is formulated in detail for language-related ERPs in Bornkessel-Schlesewsky and Schlesewsky (2019). Overly simplified (see Friston, 2005, for mathematical details), the predictive coding framework rests on the assumption that the (human) brain actively creates explanations for the causes of its own sensory inputs (e.g., Friston, 2005). The brain achieves this *via* an internal, hierarchically organized generative model of the world, thereby constantly mapping (hidden) causes to sensory consequences (predictive coding). This internal model is constantly checked against actual sensory input (hypothesis testing). When there is a mismatch between the internal model and the sensory input, prediction error arises, leading to an instant update of the internal model. Predictive coding and hypothesis testing occur at multiple, hierarchically organized levels, starting with low levels with short timescales at which very precise predictions are generated (in language, e.g., the level of individual phones), to higher-order (conceptual) levels with increasingly imprecise, more general (“conceptual”) predictions (e.g., word meaning, sentences, and discourse structure). Within the framework proposed by Bornkessel-Schlesewsky and Schlesewsky (2019), the N400 and other language-related negativities reflect prediction error at different levels of linguistic representation, while positivities, such as the P300/P600, are related to attentional gain control (see Sassenhagen et al., 2014; Sassenhagen and Bornkessel-Schlesewsky, 2015, for further discussion). Here, we focus on the P300 and N400 ERP components because of their relevance in language processing (cf. Roehm et al., 2007; Kutas and Federmeier, 2011).

Event-related potential studies have provided empirical evidence for the relevance of prominence information in linking anaphoric expressions to a referent in discourse during online language comprehension. The most well-researched ERP component in this respect is the N400, a vertex-negative component of the human ERP, peaking at roughly 300–500 ms after the onset of a stimulus with a posterior maximum. Often interpreted as a specific correlate of linguistic meaning processing, the N400 rather reflects activity in widely distributed, heavily interacting neural networks underlying the comprehension of meaning in general (cf. Kutas and Federmeier, 2011). In the discourse literature, N400 effects are, for example, reported in replications of the repeated name penalty using ERPs (Swaab et al., 2004; Camblin et al., 2007; Ledoux et al., 2007; Almor et al., 2017). Camblin et al. (2007), for instance, found increased N400 amplitudes following repeated name anaphors as compared to pronoun anaphors. This effect was absent, when a repeated name referred to a referent that formed part of a conjoined phrase (e.g., “John and Mary went to the store, so that John …”). Results of a study by Schumacher et al. (2015) point into the same direction. They contrasted ERPs following personal pronouns and demonstrative pronouns in German (e.g., “Der Feuerwehrmann will den Jungen retten …, aber er/der hat” and “The firefighter wants to rescue the boy …, but he/Dem has …”) and found more pronounced N400 amplitudes following demonstrative pronouns, as compared to personal pronouns. They attribute this effect to differences in the form-to-function mapping of the two types of expressions: While personal pronouns are highly expected and single out the most prominent referent in a discourse model (which is considered the ideal referent), demonstrative pronouns explicitly exclude coreference with the most prominent referent. According to the authors, this additional information (“Exclude the default referent!”) is reflected in an increase in the N400 component. Streb et al. (2004) reported an N400 for increased distance (measured in sentences) between anaphor and antecedent. Similar results have been reported with regard to various linguistic prominence features, for instance, givenness (Burkhardt, 2006; Schumacher and Hung, 2012), topicality (Hung and Schumacher, 2012; Wang and Schumacher, 2013), animacy (Nieuwland and van Berkum, 2006; Hung and Schumacher, 2012), or parallel structure/role (Streb et al., 1999). Overall, the N400 in referential processing can be considered to reflect a mismatch with regard to prominence-based predictions.

ERP studies of referential processes frequently also report a late positivity (P600) following the N400 which is usually interpreted as a correlate of revision in a wide sense (van Berkum et al., 1999; Schmitt et al., 2002; Hammer et al., 2005; Burkhardt, 2006; Lamers et al., 2006; van Berkum et al., 2007; Hammer et al., 2008; Schumacher, 2009; Brouwer et al., 2012; Hung and Schumacher, 2012; Schumacher and Hung, 2012; Schumacher et al., 2015). In Schumacher et al. (2015), for instance, the N400 effect for demonstrative pronouns is followed by a late positivity effect. They interpret the effect as reflecting updating processes associated with the demonstrative pronoun’s referential shift potential. As argued elsewhere (Coulson, 1998; Coulson et al., 1998; Sassenhagen et al., 2014; Sassenhagen and Bornkessel-Schlesewsky, 2015; Brilmayer et al., 2017), we view the P600 as a P300 shifted in latency. There is evidence that highly predictable (i.e., preferred) linguistic input leads to an earlier peaking P300. For instance, Roehm et al. (2007) compared the ERP following sentence final words with a cloze probability near one (antonyms, e.g., “white” in “The opposite of black is white.”) with related (“The opposite of black is yellow.”) and unrelated words (“The opposite of black is nice.”). They found a gradient P300 effect (antonym, related, and unrelated) within the time range of the N400, suggesting an overlap of these components. The authors argue that this P300 reflects a prediction match response, or, more precisely, the absence of prediction error following highly predictable linguistic input. In other words, the meaning of the antonym is already predicted and integrated before the word is encountered. If, as it was the case in the related and unrelated condition, there occurs prediction error with regard to linguistic meaning, the same biphasic N400-P600 pattern as in Schumacher et al. (2015) can be observed. As discussed in Alday and Kretzschmar (2019), the N400 and P300 both seem to be sensitive to prediction, yet while the N400 reflects the processing of stimulus related features (e.g., linguistic prominence features) necessary for categorization, the P300 reflects the categorization process itself. Accordingly, in the absence of prediction error, no further information is needed for stimulus classification, hence the early P300 in Roehm et al. (2007), while with linguistic prediction error, and hence, categorization uncertainty, an N400 arises, reflecting the processing of stimulus features relevant for categorization (“evidence accumulation”). The P300 in turn reflects the categorization process itself, thereby linking perception and (cognitive) (re-)action (cf. Verleger et al., 2005). If we transfer this to referential expressions and the establishment of anaphoric relations, prominent referents (e.g., agents/subjects) are predicted to be continuous in discourse and to be referred to by a personal pronoun. When the pronoun is encountered, the referential relation is already established, since it was predicted, similar to the antonyms in the study by Roehm et al. (2007). Hence, we expect a critical involvement of the P300 in the establishment of referential relations in the present study, especially following personal pronoun anaphors.

Moreover, from this perspective, the N400 associated with the repeated name penalty reflects a mismatch between the predicted referential form (pronoun) and the detected referential form (name). Along these lines, the N400 in Schumacher et al. (2015) reflects a mismatch between the predicted referential form (personal pronoun) and the detected form (demonstrative pronouns), while the positivity indicates attentional reorientation toward the non-prominent referent. In other words, the late P300 in referential comprehension reflects the linking of an unpredicted referential form to an unpredicted antecedent in memory (i.e., its categorization) and its potential consequences for discourse (i.e., referential shift). Burkhardt (2006) investigated different degrees of givenness (coreferential expression vs. inferred expression) and also reported a biphasic pattern: The N400 for inferred expressions reflects a mismatch between the predicted entity and the detected entity, and the positivity represents reorientation toward a new referent.

Evidence for an involvement of an “early” P300 in the processing of referential expressions stems from Brilmayer et al. (2019). In this ERP study using an audio book recording of The Little Prince by de Saint-Exupéry (2012), the authors contrasted pronouns of the first, second, and third person singular with reference to the main character (The Little Prince) or his interlocutors. They found an early peaking positivity (200–300 ms) that was sensitive to linguistic person, indicative of attentional processes. First person pronouns thereby elicited the most positive going amplitudes, followed by third person and second person pronouns. Interestingly, the P300 was insensitive to referent identity, suggesting that early processes driven by linguistically definable features already occur in early time windows preceding the N400. Since we use the same data set in the present study, we expect effects in this time range to occur in our analysis. Before we move on to the experimental methods, we would like to discuss several aspects related to naturalistic designs.

In psycho- and neurolinguistic research, a growing interest in speech and language comprehension under naturalistic conditions is observable (e.g., Schmitt et al., 2002; Brennan and Pylkkänen, 2012; Willems, 2015; Alday et al., 2017; Mak and Willems, 2018; Sassenhagen, 2018; Bhattasali et al., 2019; Brennan et al., 2019; Brilmayer et al., 2019; Schilling et al., 2021). Linguistic research thereby follows a more general trend in the cognitive neurosciences toward a more “realistic” picture of brain processes as they occur during real-life events (cf., for instance, Schilbach et al., 2013) (M) EEG higher-order language studies with naturalistic stimuli are still rare, use auditory short stories as the preferred stimulus type, and span a wide variety of topics: predictive sentence comprehension in participants with autism spectrum disorder (Brennan et al., 2019), syntactic structure building (Brennan and Pylkkänen, 2012; Brennan et al., 2016), lexical frequency (Sassenhagen, 2018), pronouns and linguistic person (Brilmayer et al., 2019), thematic role, case, and syntactic function (Alday, 2019), and content versus function words (Schilling et al., 2021). This diversity makes a direct comparison of the results difficult. Yet, there are commonalities all these studies that are compatible with results of controlled experiments: Alday (2019) found an N400 effect (300–500 ms) associated with thematic role, Sassenhagen (2018) and Alday (2019) report an N400 effect associated with lexical frequency, and Brennan and Pylkkänen (2012) as well as Schilling et al. (2021) provide evidence for an involvement of the N400 in naturalistic language comprehension. This suggests that certain generalizations derived from controlled experiments, in particular the ubiquity of the N400, can serve as a useful starting point for hypotheses generation with naturalistic designs.

One of the great challenges of naturalistic stimuli is that ecological validity (“naturalness”) and experimental control are two extremes on a continuum, so that a gain in one leads to a loss in the other (cf. Willems, 2015). Audio book stimuli, such as the present recording, contain a vast amount of variance outside of experimental control. Besides linguistic variables, such as case, syntactic function, or word order, the speech signal itself is a critical source of variance: differences in formant pitch between words, differences in intensity or duration (e.g., in the present audio book, word durations range from 30 to 2.6 s). The traditional (grand-)averaging method cannot adequately model this variance (see Alday, 2019, for discussion). Here, we therefore follow a more adequate approach to the analysis of EEG data from naturalistic experiments that is based on the linear model and allows the consideration of continuous covariates in the statistical analysis (Sassenhagen and Alday, 2016).

The probably most well-known approach of this kind is the linear regression-based approach to event-related potentials (Smith and Kutas, 2015a,b; Ehinger and Dimegen, 2019). Other than traditional averaging, the rERP framework rests on the assumption that every sample of an EEG signal can be described as the linear combination of different factors with different weights (*β*-coefficients), i.e., by the linear model. In its mass univariate formulation (cf. Hauk et al., 2006), epoched EEG data are modeled *via* separate linear models for every sample point. For instance, for epoched data from −200 to 800 milliseconds time-locked to a critical word with a sampling rate of 500 Hz, 500 linear models would be calculated, resulting in 500 *β*-coefficients per factor of interest, one for each sample. These coefficients (or the fitted values) can be treated just like traditional ERPs, for instance for (second-order) statistical analyses. One of the big advantages of this method thereby is that continuous covariates and categorical variables of interest can easily be accounted for within a single model. As noted by Smith and Kutas (2015a), in a perfectly controlled design, the rERP and the ERP approach would yield identical results. With naturalistic stimuli, however, there are considerable differences between the results of traditional grand-averaging and regression-based approaches, because of the uncontrolled variance of the stimulus material of interest. Using the mass univariate rERP method, we are able to separate the brain responses to these (linguistic) nuisance variables from those that are related to the variables of interest (the form of anaphor and antecedent). At this point, it is important to note that we do not use the linear deconvolution approach described in Smith and Kutas (2015a,b). Therefore, we have to keep in mind that our results still contain overlapping brain responses to adjacent words, especially in the baseline interval and at latencies of the late components of the rERP (<400 ms). Different variants of the rERP method have already been successfully applied to linguistic experiments in the visual (e.g., Hauk et al., 2006) and auditory domain (e.g., Brennan and Pylkkänen, 2012; Sassenhagen, 2018; Alday, 2019; Röhr et al., 2020; Ventura et al., 2020).

In the following study, we aim at exploring the form-function relation between anaphors and their antecedents as outlined above. Prior to any analysis steps, we chose to analyze the P300 (200–300 ms) and N400 time windows (300–500 ms), because of the sensitivity of the P300 to predictability in language comprehension in general (e.g., Roehm et al., 2007; Sassenhagen et al., 2014; Brilmayer et al., 2017; Alday and Kretzschmar, 2019) and because of the effects in Brilmayer et al. (2019), who recorded the data used for the present analysis. The N400 was chosen because of the ubiquity of N400 effects in discourse research and language research in general (cf. Kutas and Federmeier, 2011). Based on previous findings on the P300 and N400 component in language comprehension, we expect P300 and N400 amplitude to be sensitive to the prominence (i.e., predictability) of a referential form. In particular, we expect the P300 amplitude to increase along the prominence scale provided above (pronoun-pronoun chain > noun-pronoun chain > noun-noun chain > pronoun-noun chain). For the N400, we expect the most pronounced mismatch effect for the pronoun-noun chain relative to the noun-noun chain, because given the form-function mapping, the former combination is the least predicted (pronoun-noun chain > noun-noun chain); as far as pronoun anaphors are concerned, both antecedent types license pronominal coreference, and hence, no prediction error might arise, but alternatively, the pronoun-pronoun chain might represent the most ideal referential chain (noun-pronoun chain ≥ pronoun-pronoun chain).

## Experiment

### Materials and Methods

#### Participants

In the present study, the data of 35 participants were analyzed, all participants were monolingual native speakers of German (23 females; mean age: 25.0 years, range 20–34) with normal hearing and unimpaired vision was analyzed. The data of 25 participants (14 females; mean age: 24.4 years, range 20–29) stem from a study by Brilmayer et al. (2019); 10 additional participants were recorded in our own laboratory (nine females; mean age: 25.6 years, range 21–34). Participants received either course credit or monetary compensation for participation. The data of three participants had to be excluded from further analysis due to heavy artifact contamination.

#### Experimental Stimuli and Procedure

A German audio book version of The Little Prince by Antoine de Saint-Exupéry (recording by Will Quadflieg, chapters 1–15, excluding chapters 5, 6, and 14) served as experimental stimulus. The book contains non-dialog passages written from the perspective of a third person narrator who is also a protagonist in the story, and dialog passages in which the main protagonist, The Little Prince, interacts with a variety of characters. Dialog passages make up ~40.8% of the story (58.2% narrative passages). The rest of the text consists of free indirect discourse, indirect speech, and direct thought (~1%). The recording was segmented using automatic speech segmentation provided by the Munich Automatic Segmentation (MAUS) Web interface (Schiel, 1999; Kisler et al., 2016), combined with manual corrections.

For the present study, we restricted our analysis to personal pronouns and nouns that were encoded as grammatical subject with a noun or pronoun antecedent, including also pronouns in direct speech and all other types of discourse, resulting in a total of 215 pronouns [63 with noun antecedent; ich, “I”; du, “you.sg,” er, sie, es, “he, she, it,” wir, “we,” Ihr, “Your” (hon.), Sie, and “you” (hon.)] and 91 nouns (40 with noun antecedent and 29 different nouns, e.g., Geograph, “geographer,” Prinz, “prince,” Blume, “flower,” Planet, and “planet”). We chose these restrictions, in order to reduce the amount of uncontrolled variance in our data and to increase the reliability of our statistical analyses.

In our analysis, we compare ERPs following anaphoric expressions based on their referential type (noun/pronoun; anaphor type) and that of their antecedent (antecedent type). The resulting four referential chains have already been exemplified in (1–4). As argued above, we focus here on the P300 (200–300 ms) and N400 time window (300–500 ms). The distribution of anaphor types and antecedent types in the sample used for the rERP analysis is listed in Table 1 (values in parentheses represent the occurrences in the entire audio book).

**Table 1:** Distribution of referential types in the analyzed sample and the whole audio book recording (in parentheses).

	Noun antecedent	Pronoun antecedent
Noun anaphor	40 (114)	51 (81)
Pronoun anaphor	63 (88)	152 (201)

Table 2 summarizes the distribution of anaphors and antecedents with regard to the prominence lending features *identity of the referent* (prince, interlocutors, and other), *syntactic function* (subject, direct object, and other), and *definiteness* (definite, indefinite, and other). In summary, reference to either The Little Prince or interlocutors of The Little Prince made up ~72% of all referents in the current sample. Crucially, although the form (definite, indefinite, and proper name) of the noun anaphors varied, ~82% of them were definite. We find a very similar pattern for the antecedent expressions: About 75% of them were grammatical subjects (nouns: ~67%, pronouns: ~80%), and 90% were definite (nouns: ~74%, pronouns: >99%). That is, about four-fifth of antecedents were definite, grammatical subjects, although we did not formulate any selection criteria regarding their linguistic features. It seems that selecting only subject anaphors already filtered a great amount of linguistic variance among the antecedent expressions.

**Table 2:** Distribution of several prominence features of anaphors and antecedents in the analyzed sample (referent identity, definiteness, and syntactic).

Anaphor				Antecedent					
		Noun (91)	Pronoun (215)			N-N (40)	N-P (63)	P-N (51)	P-P (152)
Referent	Prince	25	85	Referent	Prince	9	25	16	60
	Interlocutors	39	68		Interlocutors	13	25	25	43
	Other	27	62		Other	18	13	10	49
Definiteness	Definite	75	213	Definiteness	Definite	28	48	50	151
	Indefinite	8	0		Indefinite	9	12	0	0
	Other	8	2		Other	3	3	1	1
				Syn. function	Subject	25	44	36	127
					Direct object	8	12	10	8
					Other	7	7	5	17

Note that syntactic function is not listed for anaphors, since *via* our selection criterion, all anaphors were syntactic subjects. N-N = noun antecedents of noun anaphors; N-P = noun antecedents of pronoun anaphors; P-N = pronoun antecedents of noun anaphors; and P-P = pronoun antecedents of pronoun anaphors.

As mentioned in the introduction, Givón (1983) introduced referential distance and persistence as measures of textual cohesion and we thus annotated the present audio book recording for these quantitative prominence measures. Referential distance counts the number of clauses between a referential expression and the last mention of its antecedent. It ranges from 0 (same clause, e.g., reflexives, such as “John shaved himself.”) to a maximum of 20, which is also the ceiling value assigned to newly introduced referents. Persistence determines the number of clauses in which a referent recurs in subsequent discourse. It can take any full number starting with 0 (no recurrence). In the following, we summarize these results, since referential distance and persistence entered the Principal Component Analysis detailed below (see also Torregrossa et al., 2018).

**Figure 1:**
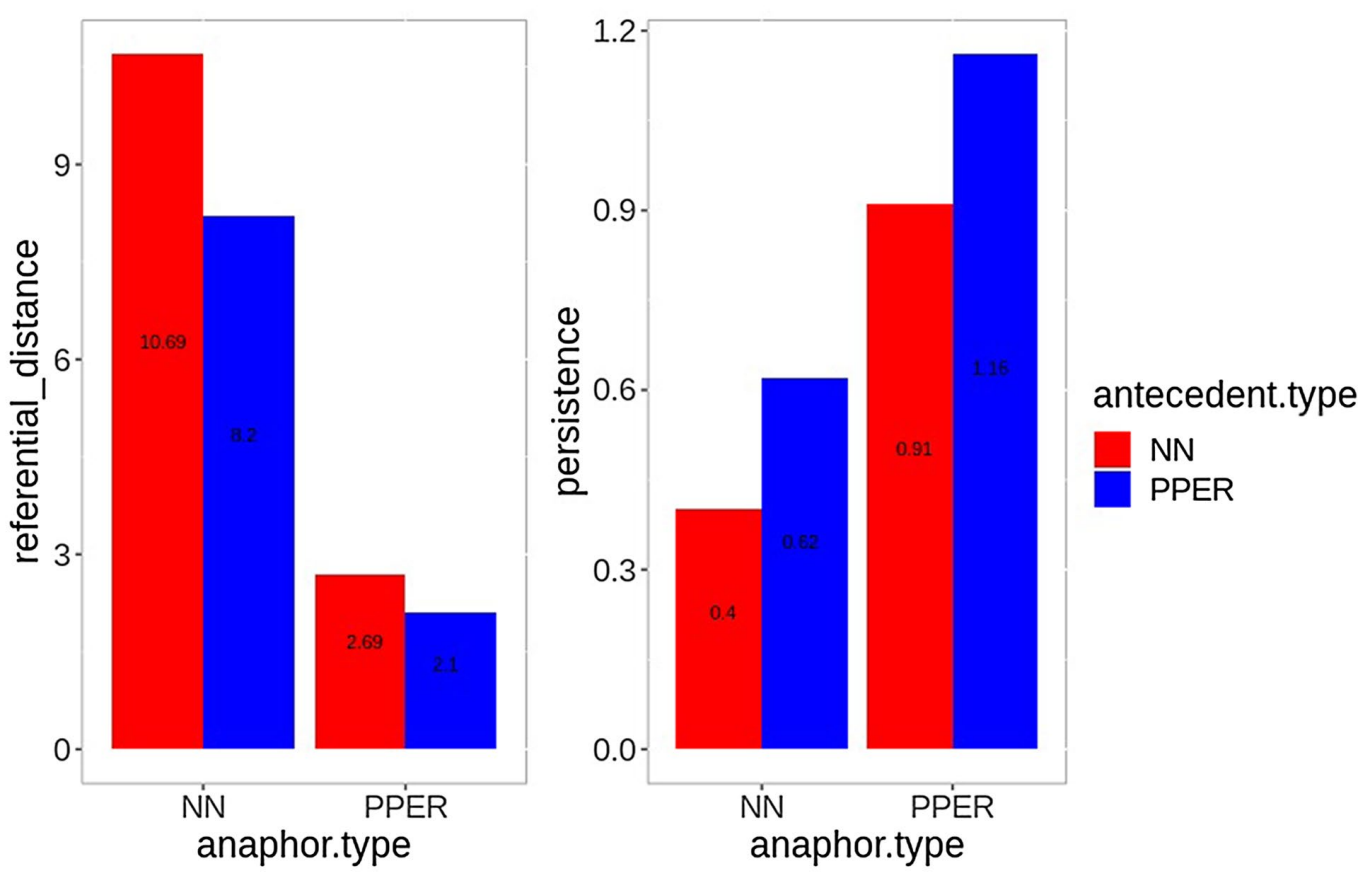
Mean referential distance (left) and persistence (right) for all noun (red) and pronoun (blue) anaphors in the audio book recording by antecedent type.

Figure 1 presents the results. As the left panel shows, noun anaphors are generally further away from their antecedent (10.69 and 8.2 clauses for noun and pronoun antecedents, respectively) than pronoun anaphors (2.69 and 2.1 clauses for noun and pronoun antecedents, respectively). In general, this pattern is consistent with the common observation that pronouns are usually closer to their antecedent than nouns (e.g., Givón, 1983; Gundel et al., 1993).

The difference between antecedent types for noun anaphors (+2.76 clauses for noun antecedents) is, thus, more pronounced than for pronoun anaphors (+0.59 clauses). With regard to persistence (right panel), we can observe the opposite pattern. Noun anaphors with noun antecedent have the lowest persistence value (0.4 clauses), followed by nouns with pronoun antecedent (0.62 clauses), pronouns with noun antecedent (0.91 clauses), and pronouns with pronoun antecedent, which have the highest persistence value (1.16). Interestingly, the prominence ranking resulting from the referential distance and persistence values (pronoun-pronoun chains > noun-pronoun chains > pronoun-noun chains > noun-noun chains) is not identical with the prominence ranking based on referential form, as the order of noun-noun and pronoun-noun anaphors is reversed. However, the results of the text analysis demonstrate nicely that discourse structural properties of referential expressions do not only depend on the referential form of the anaphor, but that it interacts with the referential form of the antecedent. It is therefore crucial to consider both in an analysis.

#### EEG Recording and Analysis

The scalp EEG was recorded using 32 Ag/AgCl electrodes attached according to the international 10–20 system using an elastic EEG cap (EasyCap, EasyCap GmbH, Herrsching, Germany). The EEG was recorded and digitized with a sampling rate of 500 Hz relative to right mastoid reference (BrainAmp DC, Brain Products, Gilching, Germany). Impedances were kept under 3 kΩ. The data were analyzed using a python3 implementation of MNE python (Gramfort et al., 2014) version 0.19. Before any further preprocessing procedures, experimental pauses were manually removed from the raw recordings. Afterward, the data were re-referenced to linked mastoids. We then used independent component analysis (ICA) for artifact correction. For ICA, the EEG was filtered with a 1 Hz high-pass filter in order to approach stationarity and a 45 Hz low-pass to remove line noise. ICA was then computed with a decimation factor of 4. Afterward, artifact components (blinks and saccades) were selected and removed from the unfiltered EEG, to which the IC solution was applied. Instead of applying a baseline correction, we chose to filter the EEG with a 0.3 Hz high-pass and a 30 Hz low-pass filter (cf. Friederici et al., 2000; Wolff et al., 2008; Widmann et al., 2015; Maess et al., 2016a,b). For the calculation of the regression-based ERPs (rERP), we re-sampled the data to 250 Hz in order to reduce computational demands.

#### Principle Component Analysis

As mentioned above, naturalistic stimuli contain huge amounts of uncontrolled variance. Since the available data and thus statistical power are limited (the inclusion of more than three covariates leads to problems with overfitting), we had to decide which of the available covariates to include in the regression model. We thus used principal component analysis to determine which of the available covariates (mean f0-pitch, mean intensity, frequency class, word duration, referential distance, and persistence) explain the most variance in the present sample of the audio book version of The Little Prince. Of the six variables, we chose the three variables with the highest contribution and quality of representation in the first three dimensions (~60% of 72% of total variance): mean f0-pitch, duration, and referential distance. It is important to note here that referential distance was actually outranked by frequency class. The reason why we still chose referential distance over frequency class lies in its distribution: Frequency class almost perfectly divides the data into noun and pronoun anaphors. While 86.5% of pronouns have a frequency class at or below the mean frequency class of about 6.8 (mean: 4.1, classes 3: 40%, 4: 46.5%, and 8: 13.5%), 100% of noun anaphors lie above it (mean: 13.2, range: 7–21). With regard to referential distance, pronouns still have a lower mean than noun anaphors (2.3 vs. 7.8 sentences), yet they both cover the full range from 0 (antecedent in the same sentence) to 20 (20 sentences or more to antecedent, or newly introduced). In addition, duration and frequency class cover almost identical data points (*r* = 0.8). The inclusion of both in one model is thus of low explanatory value.

#### rERP Calculation

The rERP calculation was performed using the lm() function in R with amplitude scaled to the standard deviation scale as dependent variable and anaphor type and antecedent type as factors with interaction. Duration, referential distance, and mean f0-pitch were added as covariates without interactions. All factors were encoded using deviation coding. We calculated linear models by participant, channel, and sample (= 6526 models per participant). From each of these models, we extracted the fitted values for the interaction of anaphor and antecedent type for the second-order statistical analysis using the function effect() from the package effects (Fox and Weisberg, 2019), thereby disregarding the effects of the covariates. The resulting single-subject rERPs are comparable to traditional single-subject averages and can be used for further analysis in the same way.

#### Second-Order Statistical Analysis

The second-order statistical analysis was carried out using linear mixed-effect models as implemented in the lme4 package for R (Bates et al., 2014) with N400 (300–500 ms) amplitude as dependent variable. The model included fixed effects for antecedent type (noun/pronoun) and anaphor type (noun/pronoun), as well as two continuous topographic fixed effects based on two-dimensional electrode positions (saggitality/laterality). Contrasts were encoded using deviation coding, so that individual coefficients represent differences from the (grand) mean. Since all our factors have two levels, they are equidistant to the mean, which means that model coefficients can be directly interpreted as differences between conditions. The model was fitted using a backward approach, starting with maximally specified random effects until we arrived at a converging model (cf. Bates et al., 2015). The model included a by-participant intercept and by-participant random slopes for each fixed factor without interactions. In the following, we will only discuss contrasts that are significant *via* the |*t*| ≥ 2 criterion corresponding to traditional *p* < 0.05 (Baayen et al., 2008). To assess pairwise statistical significance, we estimated marginal means using the function emmeans() as implemented in the R library emmeans (Lenth et al., 2019).

### Results

#### rERP

**Figure 2:**
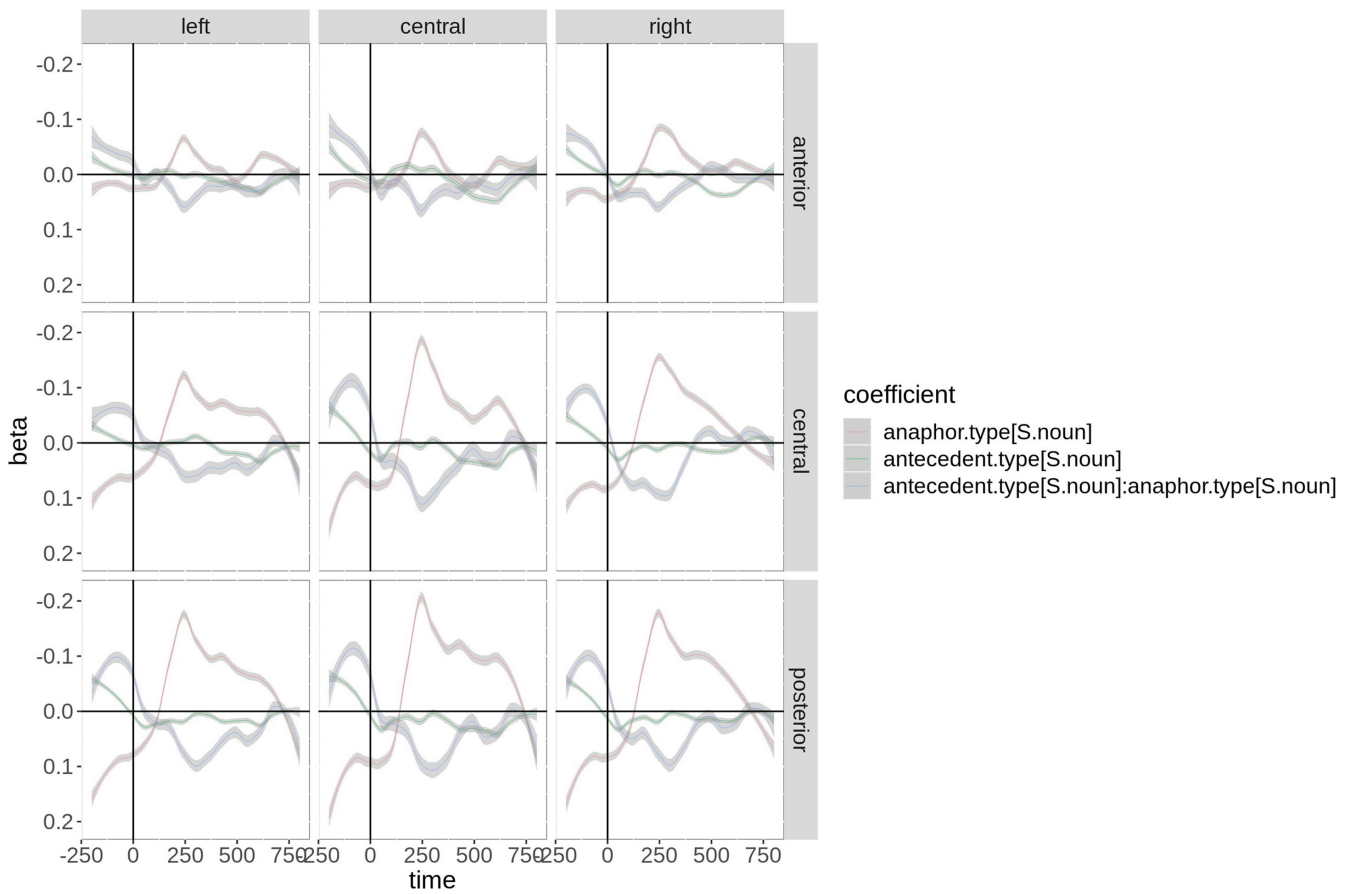
Time course of the beta coefficients of the critical predictors and their interaction by region-of-interest (ROI). For plotting purposes, the continuous topographic variables were grouped into ROIs based on two-dimensional coordinates. Shaded areas represent 83% confidence intervals (an approximation to the traditional 0.05 level of significance for visualization purposes).

**Figure 3:**
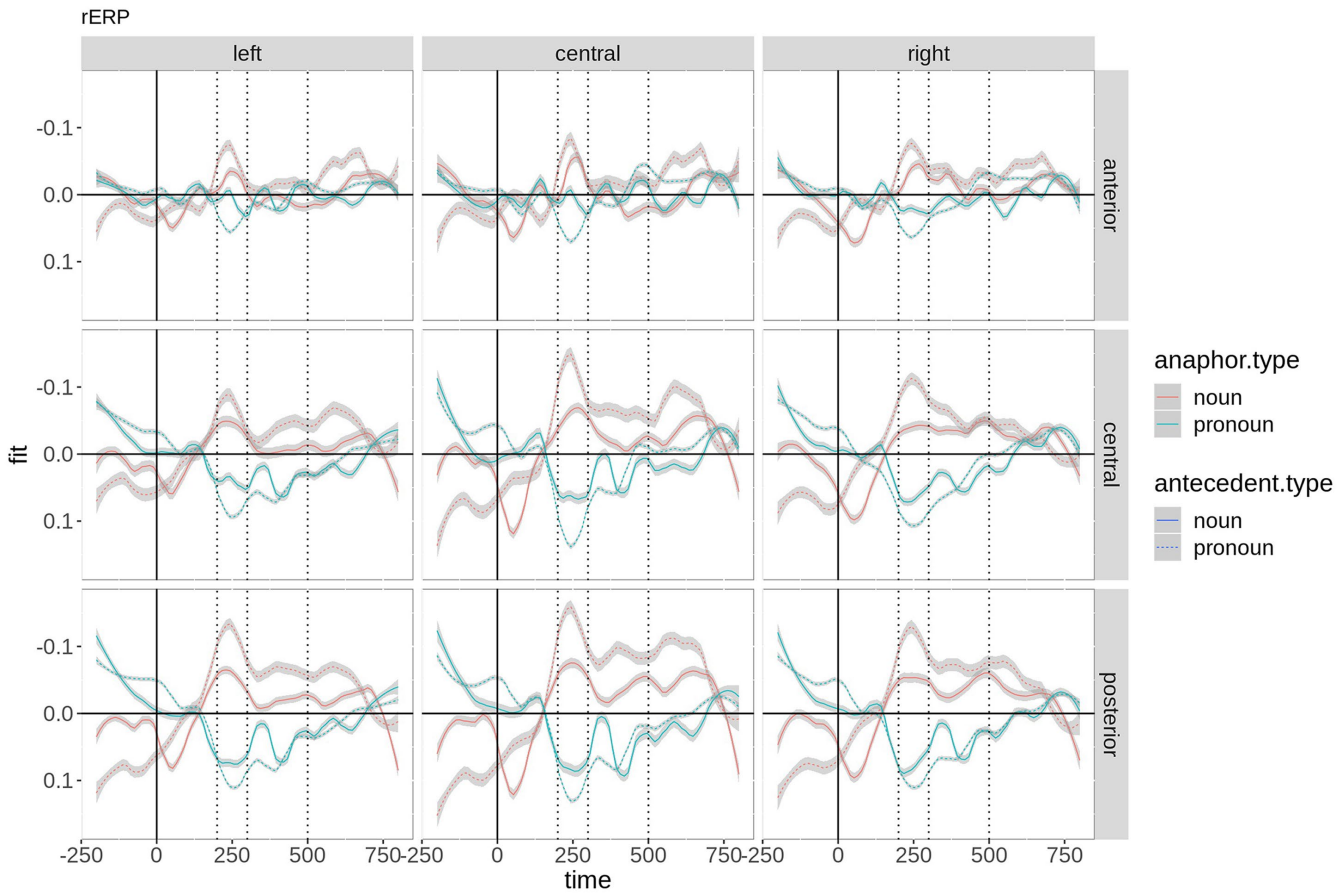
Time course of the beta coefficients of the critical predictors and their interaction by ROI. For plotting purposes, the continuous topographic variables were grouped into ROIs based on two-dimensional coordinates. Shaded areas represent 83% confidence intervals (an approximation to the traditional 0.05 level of significance for visualization purposes).

Figure 2 shows the beta coefficients of the critical predictors and their interaction by region-of-interest. Although the coefficients start with a large offset in the baseline interval, anaphor type and its interaction with antecedent type show a zero crossing (reversal of the sign), suggesting that they are strong predictors. Antecedent type has almost no effect (beta coefficient very close to zero), although a small positive effect is visible at ~400 + ms. Anaphor type (red) thereby shows a negative effect over central and posterior electrodes in the time window of the N400. The interaction shows the strongest effect between ~200 and 350 ms. Since the beta coefficient of an interaction is complicated to interpret, the fitted microvolt values are plotted in Figure 3. First, the difference between anaphor types becomes obvious: The rERP of pronoun anaphors is characterized by a positivity with posterio-central distribution, while the rERP of nouns is characterized by a posterio-central negativity. Yet, as discussed above, this difference can only be interpreted with caution, since nouns and pronouns differ critically in their phonetic properties and temporal extent (in the current recording, nouns are on average 2.9 times longer than pronouns: 430 ms vs. 150 ms). Therefore, we focus on the effects of antecedent type within anaphor types.

Within noun anaphors (Figure 3; red), chains with pronoun antecedent elicit more negative going amplitudes peaking at ~250 ms distributed over the entire scalp with a posterior maximum, and between ~400 and 750 ms (i.e., within the N400 time window) over central and posterior electrodes. Within pronoun anaphors (Figure 3; blue), chains with pronoun antecedent elicit more positive going amplitudes at left central and posterior electrodes between ~200 and 400 ms as compared to pronoun anaphors with noun antecedent. This difference is most pronounced at ~250 ms after word onset. In the following sections, we report the results of the time-window analysis.

#### Second-Order Statistical Analysis

##### P300

The results of the statistical analysis of the P300 time window are summarized in Table 3. We focus here on the effects involving the critical factors antecedent type and anaphor type with |*t*| > = 2. As follows from Table 3, there is a significant main effect of *anaphor type*, significant two-way interactions saggitality*antecedent type, laterality*anaphor type, saggitality*anaphor type, and antecedent type*anaphor type. In addition, the model includes a significant three-way interaction between *saggitality, antecedent type*, and *anaphor type*. We focus here on the significant contrasts that are not part of a higher-order contrast, i.e., laterality*anaphor type and saggitality*antecedent type*anaphor type. To assess statistical significance, we estimated marginal means using the function emmeans() as implemented in the R library emmeans (Lenth et al., 2019). For this purpose, we split the continuous topographic variables laterality and saggitality into three bins: a central bin (lateral and central midline) and two bins based on the mean of their positive (right/anterior) and negative values (left/posterior). According to this analysis, the contrast between noun and pronoun anaphors is significant at left (estimate = −0.125, *t* = −6.40, *p* < 0.001), central (estimate = −0.133, *t* = −6.87, *p* < 0.001), and right electrodes (estimate = −0.141, *t* = −7.21, *p* < 0.001).

**Table 3:** Summary of the statistical model of the P300 time window.

Coefficient	*β*	*SE*	*t*
(Intercept)	−0.002	0.012	−1.79
Laterality	0.003	0.002	1.28
Saggitality	0.007	0.003	2.81
Antecedent type (noun)	−0.005	0.006	0.77
Anaphor type (noun)	−0.068	0.009	−7.16
Laterality:saggitality	0.002	0.005	0.33
Laterality:antecedent type (noun)	0.002	0.002	0.79
Saggitality:antecedent type (noun)	0.011	0.003	4.31
Laterality:anaphor type (noun)	−0.005	0.002	−2.80
Saggitality:anaphor type (noun)	0.049	0.003	−19.8
Antecedent type (noun):anaphor type (noun)	0.020	0.005	4.05
Laterality:saggitality:antecedent type (noun)	−0.002	0.005	−0.38
Laterality:saggitality:anaphor type (noun)	0.009	0.005	1.85
Laterality:antecedent type (noun):anaphor type (noun)	−0.000	0.002	−0.12
Saggitality:antecedent type (noun):anaphor type (noun)	0.006	0.003	2.24
Laterality:saggitality:antecedent type (noun):anaphor type (noun)	0.003	0.005	0.55

**Figure 4:**
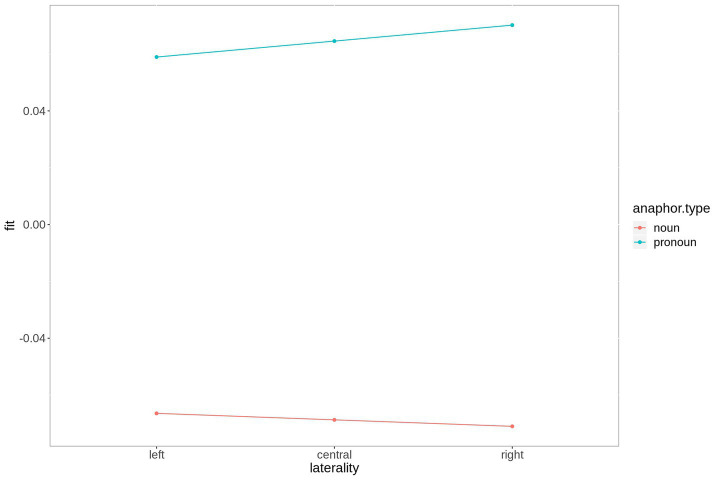
Fitted values for the interaction laterality*anaphor type in the P300 time window. For plotting purposes, the continuous variable laterality was grouped into ROIs based on 2-dimensional coordinates.

**Figure 5:**
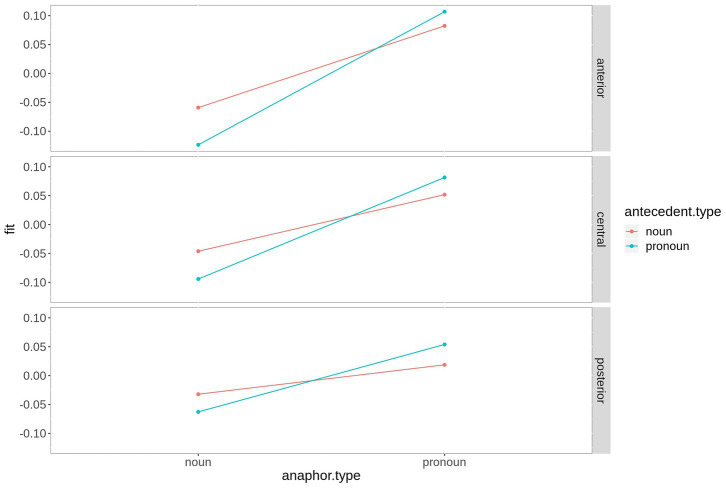
Fitted values for the interaction saggitality*antecedent type*anaphor type in the P300 time window. For plotting purposes, the continuous variable saggitality was grouped into ROIs based on two-dimensional coordinates.

The difference between the two anaphoric expressions is estimated to be largest over right hemispheric electrodes. Figure 4 contains the fitted values of this interaction. Moreover, the contrast between noun and pronoun antecedents is significant following noun anaphors at central and anterior electrodes (estimate = 0.048, *t* = 2.90, *p* < 0.007, and estimate = 0.064, *t* = 2.90, *p* < 0.001, respectively), while the contrast is significant for pronoun anaphors at central (estimate = −0.029, *t* = −2.02, *p* = 0.05) and posterior electrodes (estimate = −0.035, *t* = 2.34, *p* = 0.03). The interaction is plotted in Figure 5.

#### N400

The results of statistical analysis of the N400 time window are summarized in Table 4. It revealed a significant main effect of *anaphor type*, significant two-way interactions laterality*anaphor type, saggitality*anaphor type, laterality*anaphor type, saggitality*anaphor type and anaphor type*antecedent type. In addition, the model predicts a significant three-way interaction saggitality*anaphor type*antecedent type. Similar to the analysis of the P300 time window, we resolved the highest-order interactions using *emmeans*(), i.e., the interactions laterality*anaphor type and saggitality*antecedent type*anaphor type. The analysis revealed a significant effect of *anaphor type* at left (estimate = −0.048, *t* = −2.75, *p* = 0.009), central (estimate = −0.058, *t* = −3.30, *p* = 0.002), and right electrodes (estimate = −0.067, *t* = −3.81, *p* < 0.001).

**Table 4:** Summary of the statistical model of the N400 time window.

Coefficient	*β*	*SE*	*t*
(Intercept)	−0.002	0.006	−0.28
Laterality	−0.008	0.002	−4.83
Saggitality	0.000	0.002	0.24
Antecedent type (noun)	0.005	0.005	1.11
Anaphor type (noun)	−0.03	0.009	−3.53
Laterality:saggitality	−0.000	0.003	−0.19
Laterality:antecedent type (noun)	−0.000	0.002	−0.29
Saggitality:antecedent type (noun)	−0.000	0.002	−0.34
Laterality:anaphor type (noun)	0.007	0.002	−3.98
Saggitality:anaphor type (noun)	0.042	0.002	−19.8
Antecedent type (noun):anaphor type (noun)	0.01	0.015	2.04
Laterality:saggitality:antecedent type (noun)	−0.005	0.004	−0.12
Laterality:saggitality:anaphor type (noun)	0.005	0.004	−1.38
Laterality:antecedent type (noun):anaphor type (noun)	−0.003	0.016	−1.71
Saggitality:antecedent type (noun):anaphor type (noun)	0.08	0.002	3.97
Laterality:saggitality:antecedent type (noun):anaphor type (noun)	−0.002	0.003	−0.55

**Figure 6:**
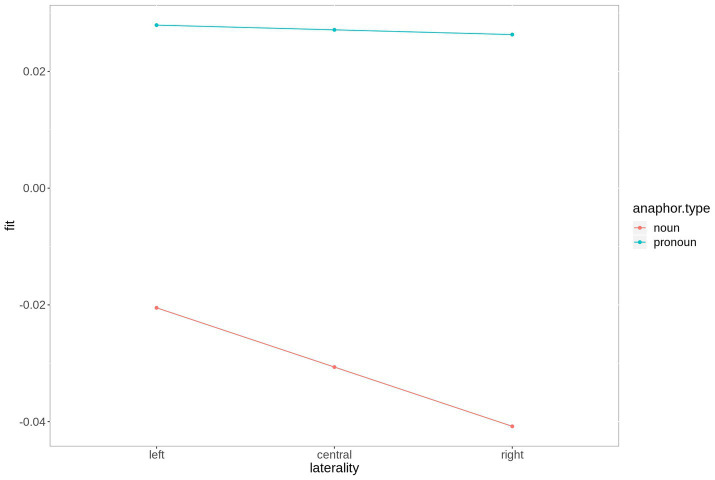
Fitted values for the interaction laterality*anaphor type in the N400 time window. For plotting purposes, the continuous variable laterality was grouped into ROIs based on two-dimensional coordinates.

**Figure 7:**
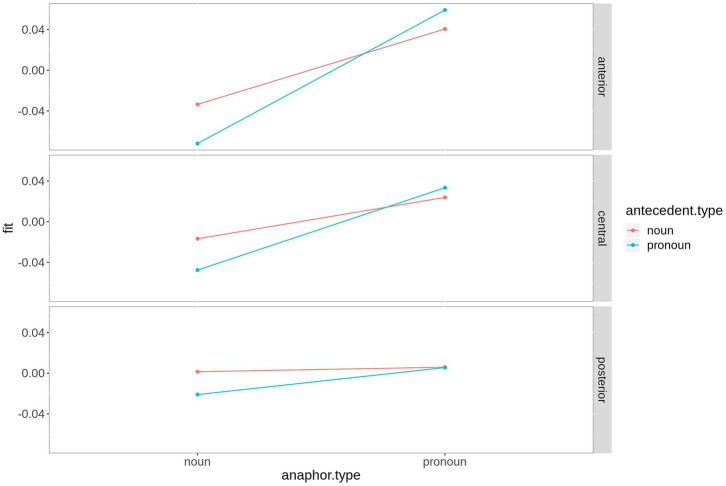
Fitted values for the interaction saggitality*antecedent type*anaphor type in the N400 time window. For plotting purposes, the continuous variable saggitality was grouped into ROIs based on two-dimensional coordinates.

As depicted in Figure 6, the effect is most pronounced over right hemispheric electrodes. Regarding the three-way interaction saggitality*antecedent type*anaphor type, the analysis revealed a significant effect of *antecedent type* at anterior electrodes for noun anaphors only (estimate = 0.039, *t* = 2.32, *p* = 0.03), while all *p*-values following pronoun anaphors exceed 0.1. The interaction is plotted in Figure 7.

#### Summary

In the present study, we compared ERP responses to anaphoric nouns or anaphoric pronouns with either a pronoun or a noun antecedent in the P300 (200–300 ms) and N400 time window (300–500 ms) using an audio book version of *The Little Prince* as experimental stimulus. The regression-based ERPs (rERP) reveal large differences in the morphology of the ERPs following noun anaphors and pronoun anaphors. While the rERP of the former is characterized by a large negative potential starting at ~200 ms after word onset, the rERP of pronoun anaphors is characterized by a positivity from ~200 ms onward. Moreover, based on the topography of the rERPs, an earlier (200–300 ms), widespread component with posterior maximum that is negative for noun anaphors and positive for pronoun anaphors can be separated from a later, sustained component only over central and posterior electrodes. Again, this component shows a negative going polarity for noun anaphors and a positive polarity for pronoun anaphors. The results of the statistical analysis in the early time window reveal a P300 amplitude gradient that follows the prominence ranking formulated above. That is, for pronouns and nouns, we found the expected gradient, both numerically, and statistically (pronoun-pronoun chain > noun-pronoun chain > noun-noun chain > pronoun-noun chain). In the N400 time window, noun anaphors elicited larger N400 amplitudes when coreferent with a pronoun than with a noun-pronoun antecedent. The N400 was not sensitive for the antecedent type of pronoun anaphors.

In summary, pronoun anaphors elicit the most positive going P300 and N400 amplitudes, yet only the amplitude of the P300 (200–300 ms) is sensitive to the type of the antecedent expression. Following noun anaphors, we also found a significant gradient that follows the prominence ranking formulated above, in both, the P300 and N400 time window.

## Discussion

The present ERP study tested the relationship of the referential form of antecedents and anaphors in referential chains and their influence on the P300 and N400 ERP components in auditory language comprehension using stimuli from a naturalistic audio book. By contrasting noun and pronoun anaphors with noun or pronoun antecedents, we hypothesized that the antecedent form is used as a predictive cue for the form of the anaphor. The results of our study are in favor of this assumption, as they reveal a significant influence of the form of the antecedent expression on the P300 and N400 amplitude following an anaphor. Most interestingly, the effects depend on the referential form of the anaphoric expression, pointing to an interaction of prediction (forward-looking function of the antecedent) and form-to-function mapping (backward-looking function) of referential expressions in the establishment of referential relations. In the following, we argue that this interaction can be explained from a predictive coding perspective on discourse comprehension.

### P300

First, let us consider the P300 time window. Recall, that, in line with the literature (e.g., Prince, 1981; Givón, 1983; Ariel, 1990; Gundel et al., 1993; von Heusinger and Schumacher, 2019), we assumed that nouns are preferably used to refer to non-prominent discourse referents, as compared to personal pronouns, which are used to refer to prominent referents in the majority of cases. Crucially, this should result in corresponding prediction match responses (enhanced P300). Following the assumption, noun anaphors with pronoun antecedent exhibit unusual (i.e., unpredicted) referential chains: A pronoun marks a referent as prominent and is used as a predictive cue for the referential form of subsequent mention. Noun anaphors, however, usually refer to non-prominent referents. A pronoun-noun chain, thus, constitutes a mismatch between the predicted form of the anaphor based on the antecedent expression and the preferred antecedent expression based on the form of the anaphor. In other words, the prediction derived from the forward-looking function of the antecedent (prominent referent > pronoun anaphor preferred) and the mapping to a referent derived from the backward-looking function of the anaphor (non-prominent referent > no pronoun antecedent preferred) contradict each other. We argue that this mismatch is visible in the attenuated P300 amplitudes following anaphors in pronoun-noun chains, reflecting the absence of a highly predictable anaphoric continuation. For noun-noun chains, by contrast (forward-looking) prediction of the antecedent (non-prominent > no pronoun anaphor preferred) and (backward-looking) form-to-function mapping of the anaphor (non-prominent > no pronoun antecedent preferred) converge, mirroring a prediction match response, as visible in amplified P300 amplitudes.

Moving on to pronoun anaphors, we found that the P300 is sensitive to the form of the antecedent expression, with higher P300 amplitudes following pronouns with pronoun antecedent, as compared to pronouns with noun antecedents. This is consistent with our hypothesis that a pronoun antecedent clearly marks a referent as prominent, which makes it a predicted (or preferred) continuous referent that is likely to be rementioned by means of a personal pronoun. The prominence information conveyed by the antecedent and the referential-form prediction derived from it are fully congruent with the prominence information of the anaphor and its preferred antecedent. That is (forward-looking) prediction of the antecedent is satisfied when the anaphor is encountered. Hence, prediction match is achieved, as visible in an increase in P300 amplitude as compared to the less prominent referents with noun antecedent. Following Alday and Kretzschmar (2019), if we wanted to provide a cognitive explanation for the P300 effect, we might say that the P300 reflects the immediate categorization of pronoun anaphors with pronoun antecedents, in the sense that they can be directly linked to a referent in the discourse model without the need for further evidence (e.g., by subsequent context). In other words, as mentioned in the introduction, we argue that pronominal reference to prominent referents is predicted to the extent that the referential relation is anticipated before the anaphoric pronoun is actually detected. With noun antecedents, this linking is more difficult, or, differently speaking, less predicted; hence, no prediction match response arises and the P300 is reduced. We attribute this difficulty to differences in prominence assigned to referents by the referential form of the antecedent expression, with noun antecedents being less prominent than pronoun antecedents.

### N400

In the N400 window, only noun anaphors show a graded N400 effect. We found that in noun-noun chains, the N400 following the anaphor was significantly reduced. We take this as evidence for the preference of nouns to corefer with a noun antecedent rather than a pronoun. By contrast, a pronoun-noun chain constitutes an exception with regard to discourse structure: An already prominent referent (realized by a pronoun) is referred to by a referential expression indicating a low level of prominence (noun). Form-to-function mapping of the anaphor (no pronoun antecedent) and the form of the antecedent (pronoun) thus contradict each other, hence the increase in N400 amplitude as a measure of a prediction error. Compared to this, a noun-noun chain is predictable, since nouns can easily be used to refer to a noun antecedent. In fact, this is quite common, for instance in referential chains consisting of an indefinite antecedent and a definite anaphor (“A man entered the room and looked around. The man then walked straight to the counter, when …”).

Following pronoun anaphors, we did not find a significant influence of antecedent type on the N400 amplitude, supporting the idea that pronoun anaphors are less dependent on the form of their antecedent. Overall, the patterns for the two time windows are thus distinct, supporting a functional dissociation between processing predicted linguistic content (leading to categorization) and encountering unpredicted linguistic content leading to prediction error. This suggests that the linguistic evidence needed for the establishment of reference might not differ between the two types of pronouns, which is reflected in the absence of an N400 effect, yet the difference in prominence might result in difficulties with respect to the categorization process.

### Comparison With Previous Experiments

The present results are compatible with previous experiments in so far as the literature on event-related potentials during referential processing consistently reports increased N400 amplitudes related to unpredicted referential relations based on prominence information (Swaab et al., 2004; Nieuwland and van Berkum, 2006; Camblin et al., 2007; Ledoux et al., 2007; Schumacher and Baumann, 2010; Hung and Schumacher, 2012; Schumacher and Hung, 2012; Wang and Schumacher, 2013; Almor et al., 2017). With the present analysis explicitly contrasting the referential form of anaphoric expressions and of their antecedents, we were able to show that prominence information based on referential form is already relevant for processing between 200 and 300 milliseconds after anaphor onset, and thus, earlier than the N400 time window usually considered crucial for referential processing. This finding is highly compatible with the results of Brilmayer et al. (2019) who provide a different analysis of the present data set. They compared pronouns of the first, second, and third person singular and found a significant P300 gradient (1 > 2 > 3) in the same early time window. As they argue, first person referents are always prominent for a variety of reasons (cf. Comrie, 1989; Dahl, 2008; Frith and Frith, 2010). These results thus corroborate the present finding that linguistic prominence information is already important at comparably early time points during the processing of referential expressions.

Crucially, the present results suggest that not only current linguistic input is reflected in this early component, but also the interaction of current linguistic input with information about the antecedent in memory. That is, stimulus-driven bottom-up information is already influenced by previous context as early as 200 milliseconds after stimulus onset. This strongly supports a predictive coding account to language-related ERPs as argued by (Bornkessel-Schlesewsky and Schlesewsky, 2019; see Bornkessel-Schlesewsky and Schumacher, 2016, for a discussion of a possible predictive coding framework for discourse comprehension). In predictive coding, top-down information from higher (conceptual) processing levels constantly influences the way in which information is processed at lower (perceptual) levels. Thus, one and the same stimulus (e.g., a personal pronoun) is processed differently based on its own prominence information and prominence information conveyed by referential forms in previous context and long-term experience. Clearly, the future research must consider such early time windows during referential comprehension, given their relevance in referential processing suggested by the present and previous studies.

Overall, our findings provide empirical support for the prominence approach to reference in discourse as proposed by von Heusinger and Schumacher (2019): Referential expressions differ in their form-to-function mapping (related to singling out, definition 1), and in the discourse predictions derived from them (related to structural attraction, definition 3). The interplay of these two functions (forward-looking) prediction and (backward-looking) form-to-function mapping, is reflected in the P300/N400 patterns following anaphoric expressions.

### Conclusion

In the present study, we showed that the P300 and N400 component are sensitive to the interaction of prominence information conveyed by an antecedent and an anaphoric expression. We showed that as early as 200 milliseconds after the onset of the anaphoric expression, the referential type of an antecedent has an influence on the ERP of an anaphor. Crucially, the effects were reversed depending on the anaphoric form. While nouns showed a graded negativity in the P300 time window (pronoun-noun chain > noun-noun chain), pronouns showed a graded positivity (pronoun-pronoun chain > noun-pronoun chain). We attribute these effects to the interaction of predictions derived from the antecedent and preferences in the form-to-function mapping of anaphors. The N400, by contrast, was only sensitive to discourse-pragmatic regularities following noun anaphors, suggesting differences in the mapping process between noun and pronoun anaphors.

## Data Availability Statement

The raw data supporting the conclusions of this article will be made available by the authors, without undue reservation.

## Ethics Statement

The studies involving human participants were reviewed and approved by the Ethics Committee of the German Linguistic Society. The participants provided their written informed consent to participate in this study.

## Author Contributions

IB is responsible for the analysis of the audio book, the preprocessing and analysis of the EEG data, as well as for statistical analysis. PBS and IB were equally involved in hypothesis generation, interpretation of the data and the writing process. All authors contributed to the article and approved the submitted version.

## Conflict of Interest

The authors declare that the research was conducted in the absence of any commercial or financial relationships that could be construed as a potential conflict of interest.

## Publisher’s Note

All claims expressed in this article are solely those of the authors and do not necessarily represent those of their affiliated organizations, or those of the publisher, the editors and the reviewers. Any product that may be evaluated in this article, or claim that may be made by its manufacturer, is not guaranteed or endorsed by the publisher.
